# Immunohistochemical Expression Patterns of CD45RO, p105/p50, JAK3, TOX, and IL-17 in Early-Stage Mycosis Fungoides

**DOI:** 10.3390/diagnostics12010220

**Published:** 2022-01-17

**Authors:** Tariq N. Aladily, Tasnim Abushunar, Ahmad Alhesa, Raneen Alrawi, Noor Almaani, Maram Abdaljaleel

**Affiliations:** 1 Department of Pathology, The University of Jordan, Amman 11942, Jordan; TSN8191036@ju.edu.jo (T.A.); alhesaahmad1990@gmail.com (A.A.); M.abdaljaleel@ju.edu.jo (M.A.); 2 Department of Dermatology, The University of Jordan, Amman 11942, Jordan; raneenalrawi@gmail.com (R.A.); nwmaani@googlemail.com (N.A.)

**Keywords:** mycosis fungoides, CD45RO, NFkB, p105/p50, JAK3, STAT3, TOX, IL-17, differential diagnosis, immunohistochemistry, dermatosis

## Abstract

The morphologic changes in early-stage mycosis fungoides (MF) might overlap with benign inflammatory dermatitis (BID). Previous studies have described altered expression patterns of several proteins in MF, but their diagnostic significance is uncertain. This study aims at examining the frequency of expression of CD45RO, NFkB-p105/p50, JAK3, TOX, and IL-17 proteins by immunohistochemistry. The cohorts included 21 patients of early-stage MF and 19 with benign BID as a control group. CD45RO was positive in all patients of MF and BID. NFkB-p105/p50 showed normal cytoplasmic staining, indicating an inactive status in all patients of both groups. JAK3 was positive in 3 (14%) MF and in 17 (89%) BID patients (*p* = 0.003). TOX was expressed in 19 (90%) and 13 (68%) patients of MF and BID, respectively (*p* = 0.120). IL-17 was detected in 13 (62%) MF and in 7 (37%) BID patients (*p* = 0.056). Co-expression of TOX and IL-17 was seen in 11 (52%) MF patients but in only 3 (16%) BID patients, which was statistically significant (*p* = 0.021). We conclude that a double expression of TOX and IL-17 may support the diagnosis of MF in the right clinicopathologic setting, while none of the immunohistochemical stains alone provided a significant discrimination between MF and BID.

## 1. Introduction

Mycosis fungoides (MF) is a mature T-cell neoplasm that arises in the skin. It is the most common type of primary cutaneous lymphoma, comprising 50–90% of skin lymphomas [1,2]. The disease is characterized by a protracted clinical course, with a stepwise progression from cutaneous patches to plaques, and eventually tumors. Disseminated disease occurs in a small subset of patients [3].

The lymphoma cells in MF tend to infiltrate the epidermis and have a special cerebriform morphology. The cell of origin of MF is believed to be a CD45RO-positive effector memory T-cell that normally resides in the skin. These cells have many subsets such as TH1, TH2, and TH17 lymphocytes, which explains the heterogeneity of the immunophenotypic characteristics of MF [3]. The genetic abnormalities in MF are numerous and complex, with no specific pattern of mutations. Examples include mutations in the tumor suppressor gene p53, CDKN2A, and CDKN2B; increased expression of NAV3, JUNB, and c-MYC; and hypermethylation of mismatch repair genes, while chromosomal aberrations are rare [4].

The diagnosis of MF in the early-patch stage can be challenging as the morphologic features are subtle and may overlap with benign inflammatory dermatitis (BID). Moreover, it is not uncommon to have multiple follow-up biopsies before reaching the definite diagnosis. The role of ancillary tests such as immunohistochemistry and molecular studies can be supportive in some patients. The aim of this study is to investigate a panel of immunohistochemical stains of the proteins involved in the pathogenesis of MF, and to explore their potential benefit in the pathologic workup. The panel includes Cluster of differentiation-45RO (CD45RO), nuclear factor kappa-light-chain enhancer of activated B-cells (NFkB)-p105/p50, Janus kinase 3 (JAK3), thymocyte selection-associated high mobility group box (TOX), and interleukin-17 (IL-17).

## 2. Materials and Methods

### 2.1. Study Group

This is a retrospective study conducted at the Department of Pathology at Jordan University Hospital, a tertiary center. After earning the approval from the Institutional Review Board at our institution, we searched the archives of surgical pathology for patients diagnosed with MF between 2013 and 2021. Patients with a confirmed diagnosis of MF by clinicopathologic correlation and who were currently on therapy were included in the study. We excluded patients with an equivocal diagnosis or with inadequate specimens. The control group was established by selecting patients with BID that were commonly encountered in clinical practice. Relative clinical data were collected such as age, gender, date of biopsy, clinical stage, duration of disease, and type of therapy. The clinical stage was established according to the criteria of the International Society for Cutaneous Lymphomas [5].

### 2.2. Morphologic Examination

Surgical specimens of the skin of MF and control groups were reviewed for adequacy. The specimens were fixed in formalin, embedded in paraffin, and processed using standard histologic methods and stained with hematoxylin and eosin (H&E).

### 2.3. Immunohistochemistry

An immunohistochemical test was performed using 4-µm thick sections of skin specimens. The slides were deparaffinized in xylene, rehydrated with graded alcohols, and heated in EDTA buffer at pH 8.0 in a steamer (Black and Decker, Towson, MD, USA). After blocking the endogenous peroxidase activity with hydrogen peroxide, the slides were washed with a buffer and then incubated with primary antibodies. The temperature and duration of incubation were set according to the manufacturer protocols. The BioGenex Autostainer (i6000 TM Diagnostic and Research) (BioGenex, Fremont, CA, USA) was used for staining. The washing buffer was 0.05 mol/L tris-aminomethane-buffered saline with 0.05% polysorbate. Diaminobenzidine was used as the chromogen. Appropriate positive and negative control samples were included. The slides were stained with antibodies specific for CD3, CD4, CD8, CD2, CD5, CD7, and CD30 (ready to use, undiluted, BioGenex); CD45RO (clone UCHL-1, 1:500, Novus Biologicals, Littleton, CO, USA); NFkB-p105/p50 (clone A2-7, 1:100, Novus Biologicals); JAK3 (clone NBP2-49629, 1:250, Novus Biologicals, TOX (clone 155768, 1:200, Abcam, Cambridge, UK); and IL-17/IL-17A (Clone 41809, 1:200, R&D system, Minneapolis, MN, USA).

The pattern of positivity was membranous for CD45RO, CD3, CD4, CD8, CD5, CD7, CD2, and CD30; cytoplasmic for JAK3, NF-kB-p105/p50, and IL-17; and nuclear for TOX. The cutoff for positivity was established when one-third or more of lymphocytes showed a positive reaction, regardless of the intensity of staining. Epidermotropic and cerebriform lymphocytes were the main focus of interpretation, as the dermal involvement of early-stage MF tends to be scanty.

### 2.4. Statistical Analysis

SPSS version 26.0 (Chicago, IL, USA) was used to analyze the results. Continuous variables were represented by means (±standard deviation), while nominal variables were described by count (frequency). Descriptive statistics were calculated for all tests and assessments of 95% confidence intervals based on binominal distribution were carried out. Fisher’s exact test was used to examine the difference between unadjusted variables. Pearson correlation test was applied to check for a positive correlation between tests. A *p*-value of 0.05 was considered significant.

## 3. Results

### 3.1. Characteristic of Patients

A total of 21 patients of MF were included. There were 13 (62%) female and 8 (38%) male patients. The range of age was 8–80 years (median: 45), including two children (8- and 12-year-old). Clinically, the skin lesions were erythematous in 20 (95%) patients and hypopigmented in 1 (5%). The lesions were predominantly plaques in 5 (24%), patches in 3 (14%), and a mixture of both in 13 (62%). One patient (5%) presented with diffuse erythroderma. The site of biopsy was from the trunk in seven (33%) patients, from the thigh in five (24%), but not documented in the remaining patients. At the time of diagnosis, 16 (76%) patients were in stage 1B; 2 (10%) in stage IIA; and 1 (6%) in each of stages 1A, IIB, and III. None of the patients showed cutaneous tumors, regional lymphadenopathy, or disseminated disease. Molecular study for T-cell receptor gene rearrangement was not available for any of the patients. Four patients (19%) were treated with topical steroids alone, 12 (57%) with topical steroids and phototherapy; 1 (5%) patient with topical steroid and methotrexate; and 2 (10%) with topical steroids, phototherapy, and methotrexate. Mild improvement of disease was documented in 20 (95%) patients, while the last patient (5%), who presented with erythroderma, showed no improvement. The duration of follow up ranged from 1–6 years (median 2). Disease progression or large-cell transformation were not reported.

The control group consisted of 19 patients diagnosed with BID. There were 12 (63%) women and 7 (27%) men, with a range of age of 10–88 years (median: 34). The diagnoses were spongiotic dermatitis in 8 (42%), lichen planus/lichenoid reaction in 4 (21%), benign lichenoid keratosis in 2 (11%), lichen nitidus in 1 (5%), pityriasis lichenoides chronica in 1 (5%), dermatophytosis in 1 (5%), fixed drug eruption in 1 (5%), and psoriasis in 1 (5%). The site of biopsy was from the upper limb in 6 (32%), lower limb in 3 (16%), trunk in 6 (32%), face in 2 (11%), scalp in 1 (5%), but not available in the remaining patients.

### 3.2. Expression Patterns of Immunohistochemical Stains

CD3, CD5, and CD2 were positive in all (100%) MF patients. CD7 downregulation was seen in one (5%) patient. A high CD4:CD8 ratio was present in 20 (95%) patients while one (5%) patient showed CD8 excess. CD30 was negative in all MF patients. In the control group, eight patients had immunohistochemical staining for T-cell antigens. All patients showed the preservation of CD3, CD2, and CD5. CD7 was lost in one patient of spongiotic dermatitis. A high CD8:CD4 ratio was seen in three patients (two spongiotic dermatitis and one lichen planus like keratosis).

**Figure 1:**
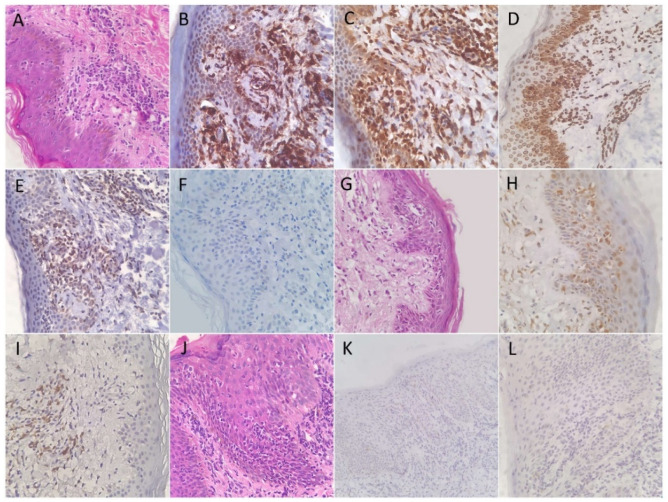
(**A**) Hematoxylin and eosin-stained histologic section from a patient with mycosis fungoides demonstrating atypical lymphocytes with enlarged hyperchromatic nuclei and clear perinuclear halos lining up the dermoepidermal junction and demonstrated epidermotropism. Most of atypical lymphocytes were positive for CD45RO (**B**) and TOX (**C**). (**D**) NFkB-P105/50 was positive in its cytoplasmic pattern, indicating an inactive form. JAK3 was also positive (**E**), while IL-17 was negative (**F**). Another patient of MF showing a positive expression of IL-17 (**G**). TOX was positive in few dermal lymphocytes but not in the epidermotropic lymphocytes (**H**). Patterns of immunohistochemical stains in mycosis fungoides (**I**). Histologic section from another patient of MF (**J**) that was negative for JAK3 (**K**) and IL-17 (**L**).

**Figure 2:**
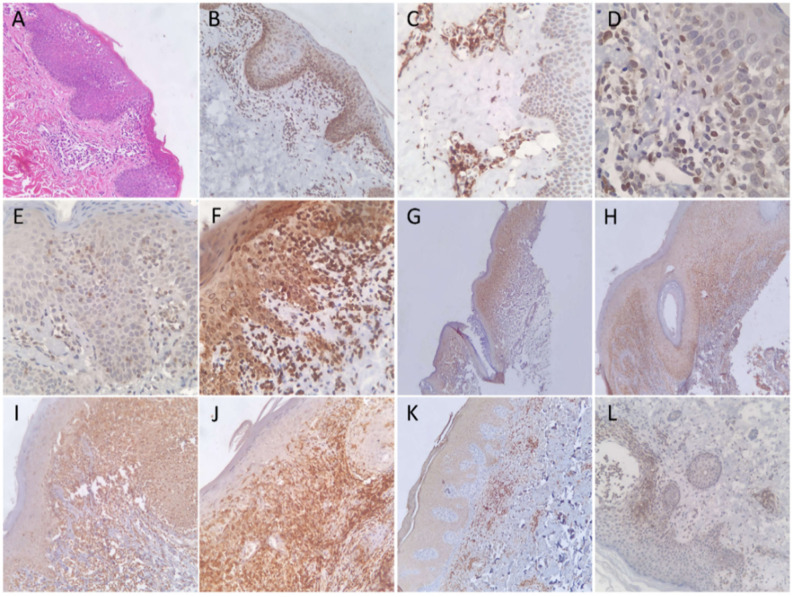
(**A**) Hematoxylin and eosin-stained histologic section from a patient diagnosed with subacute spongiotic dermatitis showing (**B**) a cytoplasmic reaction to NFkB-P105/50. The lymphocytes were positive for (**C**) CD45RO and (**D**) TOX. A patient diagnosed with psoriasis showing a positive lymphocyte reaction to (**E**) TOX and (**F**) IL-17. (**G**) Low-power view for a patient diagnosed as lichen planus demonstrating exuberant lymphocytic positivity to CD45RO, (**H**) NFkB-P105/50, (**I**) TOX, and (**J**) JAK3, while (**K**) IL-17 was negative (not shown). Another patient diagnosed as subacute spongiotic dermatitis with a positive expression of IL-17. (**L**) Another patient of spongiotic dermatitis demonstrating a positive reaction to JAK3.

CD45RO was positive in all (100%) patients of the MF and control groups. NFkB-p105/p50 showed a cytoplasmic reaction in lymphocytes of all (100%) patients of both groups, indicating an inactive form of protein. Of note, a similar pattern was seen in the adjacent squamous epithelial cells. The positivity for CD45RO and NFkB-p105/p50 was bright and diffuse in all patients. JAK3 immunohistochemical stain was positive in only three (14%) patients of MF, but in 17 (89%) of the control group. The reaction was cytoplasmic and variable in intensity among patients. TOX immunohistochemical stain was positive in 19 (90%) MF and in 13 (68%) of the control patients. The number of positive lymphocytes was variable, but tended to be high in both groups. IL-17 expression was seen in 13 (62%) MF and in 7 (37%) control patients. Figure 1 and Figure 2 show examples of the results in MF and control groups, respectively.

In the MF group, CD45RO showed the highest sensitivity. Nuclear reactions to NFkB-p105/p50 and JAK3 were not detected in any of the patients. Patients with double positivity for TOX and IL-17 was significantly (*p* = 0.021) higher in the MF (11; 52%) compared to the control group (3; 16%). The positivity to TOX and IL-17 in the MF group tended to show a positive linear trend upon correlation test, although weak (R: 0.068). Table 1 shows the sensitivity, specificity, and *p*-values of statistical difference for all of the immunohistochemical stains.

## 4. Discussion

Due to the non-specific histologic appearance of early MF, it may result in an erroneous diagnosis of an otherwise BID and possible worsening of outcome if immune suppression was used in the treatment. The classic morphologic features of early MF include proliferation of atypical cerebriform lymphocytes at the dermoepidermal junction, epidermotropism with little spongiosis, and wiry fibrosis of papillary dermis. Epidermal clusters of malignant lymphocytes around Langerhans cells, called Pautrier microabscess, represent the most robust feature of MF, but it only appears in a minority of patients at an early stage [3,6].

Immunohistochemical analysis supports the diagnosis of MF in some patients. The elevation of the CD4/CD8 ration greater than four times and the loss of CD7 in proliferating lymphocytes are the most common alterations in MF. Yet, increased histiocytes and Langerhans cells, which are normally CD4-positive, may confuse the interpretation process, and CD7 deficiency may still occur in BID. On the other hand, loss of pan T-cell markers, such as CD2, CD5, and CD3 is more specific for MF, but rarely occurs in the early stages. It is noteworthy to mention that the best assessment of immunophenotypic alterations should be at the epidermal lymphocytes, as the number of lymphoma cells in the dermis is low in early MF [7].

CD45RO is an isoform of the leukocyte common antigen with a short extracellular domain. It is normally expressed on several subsets of activated T-cells such as memory-regulatory, effector, and tissue resident lymphocytes [8]. The fraction of peripheral T-cells positive for CD45RO grows as humans age, in contrast to naïve and resting T-cells [9]. CD45RO is expressed in MF and other T-cell lymphomas [10]. Although the results of our study showed that CD45RO is a very sensitive marker of MF, it was similarly expressed in all BID patients, discounting its diagnostic value. In addition, the loss of CD45RO was described in MF by a mechanism similar to the downregulation of any other T-cell markers [11].

NFkB is transcription factor that regulates the expression of a wide range of genes involved in T-cell proliferation, survival, and cytokine production. It comprises a collection of dimers among which are p50 and its precursor p105 proteins. The pathway of NFkB is activated after interaction with tumor necrosis factor and after T-cell receptor activation, which is normally a reversible and transient process [12]. The normal cellular localization of p105/p50 proteins is cytoplasmic, which translocate to the nucleus upon activation. Constitutive activation of NFkB was described in MF and other cutaneous T-cell lymphomas, and a pharmacologic inhibitor induced apoptosis in cell lines [13]. In histologic sections, a previous study showed that 25.5% of MF patients exhibited positive nuclear staining, irrespective to the stage of disease [14]. In comparison, our study showed an inactive status of p105/p50 proteins in all MF patients, which could be explained by the relatively fewer number of examined patients and by limiting our patients to early-stage disease. Similarly, none of BID patients was positive for a nuclear stain. Thus, the NFkB proteins did not carry a diagnostic benefit for MF in our series.

The JAK3 protein belongs to the Janus kinase family of receptor-associated tyrosine kinases. It is normally located in the cytoplasm. The JAK/STAT pathway is involved in the signaling of a variety of cytokines that have been linked to the development of inflammatory disorders. It is also believed to play a key role in the evolution of cutaneous T-cell lymphomas [15]. In malignant T-cells, dysregulation of JAK3 signaling drives the expression of a variety of oncogenes, culminating in cell proliferation and resistance to apoptosis. In addition, aberrant cytokine production would modulate the tumor microenvironment and suppress immune surveillance against cancer cells. Ectopic nuclear localization of JAK3 was described in an MF cell line and in benign HIV-infected CD4+ T-cells [16]. Nuclear expression of STAT3, which is related to JAK3, was detected in 25.5% of MF patients and correlated with large cell transformation and advanced clinical stage [14,17]. None of our MF patients showed a nuclear reaction to JAK3, which corresponds to the early-stage of studied patients. In addition, cytoplasmic positivity was detected in the majority of BID. Thus, cytoplasmic JAK3 immunohistochemical positivity does not discriminate between MF and BID.

TOX is a transcription factor belonging to the high mobility group box superfamily. It is important for the differentiation of subsets of T-cells and natural killer cells. It is also implicated in tumor development as it induces CD8+ T-cell exhaustion by weakening their effector functions [18]. T-cell exhaustion occurs when cytotoxic T-cells are constantly activated, such as in cancer and chronic viral infection. TOX is overexpressed in tumor infiltrating cells and is associated with an increased expression of the inhibitory programmed-death protein. In malignant T-cell lymphomas, TOX is highly expressed in MF, T-lymphoblastic lymphoma, angioimmunoblastic T-cell lymphoma, and in a lower level in peripheral T-cell lymphoma and other lymphomas [18,19,20]. What drives TOX expression and its role in MF is unclear. The percentage of TOX-positive cells in MF increases with disease progression [21]. Our study showed that TOX expression was more common in MF than in BID, similar to previous studies [21,22]. Yet, many BID patients showed a TOX expression that could be high, and thus mere TOX positivity does not qualify for a diagnosis of MF.

IL-17 is a homodimer proinflammatory cytokine that plays an important role in host protection against infections and chronic inflammatory diseases. It is normally produced by a subset of activated CD45RO+ memory T-cells, called TH17, and interacts with its receptor on CD8+ T-cells, causing the activation of several signaling pathways. The end result is the production of inflammatory cytokines, such as tumor necrosis factor-alpha and IL-1β, which drive the infiltration of histiocytes and subsequent inflammation [23,24]. An increased level of IL-17 in skin tissues and cell lines of MF was documented in several reports using different tests [25,26]. In a previous study, malignant cells in MF were found to express IL-17 by immunohistochemistry, and it was correlated with JAK3 activation [27]. However, the authors compared the results with only three patients of BID. Our study showed that IL-17 expression was more common in the MF group, but was still detected in 37% of BID. It is noteworthy to mention that IL-17 can be normally produced by CD8+ T-cells, ϒδ T-cells, and natural killer cells, which might interfere with proper interpretation [27].

## 5. Conclusions

In summary, this study evaluated the potential immunohistochemical diagnostic benefit of a panel of proteins that were described to be overexpressed in MF. The results showed that CD45RO, NFkB-p105/p50, and JAK3 were the least helpful. Abnormal nuclear localization of NFkB-p105/p50 and JAK3, which was described previously, appears to be uncommon. In contrast, the simultaneous expression of TOX and IL-17 provided the best discrimination between MF and BID, yet remained supportive rather than definitive. We emphasize that clinicopathologic correlation remains the gold-standard way to reach the correct diagnosis of MF and, to date, no single immunohistochemical stain represents a surrogate marker for MF. A future study with a larger number of patients is recommended.

## Figures and Tables

**Table 1:** Sensitivity and specificity of each immunohistochemical stain in the diagnosis of mycosis fungoides. *p*-value indicates the statistical difference from control group.

Diagnostic Test	Sensitivity	95% CI	Specificity	95% CI	*p*-Value
CD45RO	100%	83.89–100%	0%	0–100%	1
NFkB-p105/p50	0%	0.00–16.11%	100.00%	82.35–100%	1
JAK3	14.29%	3.05–36.34%	10.53%	1.30–33.14%	0.003
TOX	90.48%	69.62–98.83%	31.58%	12.58–56.55%	0.120
IL-17	65.00%	40.78–84.61%	68.42%	43.45–87.42%	0.056

CI: Confidence interval.

## Data Availability

Reasonable data can be obtained by communication with the corresponding author.
